# Tuberculosis of the Gallbladder

**DOI:** 10.1155/2000/21392

**Published:** 2000-08

**Authors:** K. Kumar, M. Ayub, Mohan Kumar, N. K. Keswani, H. S. Shukla

**Affiliations:** 1 Department of Surgery and Pathology Institute of Medical Sciences Banaras Hindu University Varanasi 221 005 India; 2 Gorakhpur India; 3 MLN Medical College Allahabad India; 4 7 SPG Colony Lanka Varanasi 221 005 India

## Abstract

Analysis of 5 patients with gallbladder tuberculosis who had open cholecystectomy and review of literature have shown that, although still rare it presents as a part of systemic miliary tuberculosis, abdominal tuberculosis, isolated gallbladder tuberculosis and as acalculus cholecystitis in anergic patients. There are no pathognomonic signs, the diagnosis depends on suspicion of tuberculosis, peroperative findings and histological examination.


*HPB Surgery*, 2000, Vol. 11, pp. 401–404
Reprints available directly from the publisher
Photocopying permitted by license only

© 2000 OPA (Overseas Publishers Association) N.V.
Published by license under
the Harwood Academic Publishers imprint,
part of The Gordon and Breach Publishing Group.
Printed in Malaysia.

### Tuberculosis of the Gallbladder

K. KUMAR[a], M. AYUB[b], MOHAN KUMAR[a], N. K. KESWANI[c] and H. S. SHUKLA[a,*]

[a]*Department of Surgery and Pathology, Institute of Medical Sciences, Banaras Hindu University, Varanasi - 221 005,* [b]*Gorakhpur,* [c]*MLN Medical College, Allahabad*

*(Received 10 November 1999; In final form 25 January 2000)*

**Analysis of 5 patients with gallbladder tuberculosis who had open cholecystectomy and review of literature have shown that, although still rare it presents as a part of systemic miliary tuberculosis, abdominal tuberculosis, isolated gallbladder tuberculosis and as acalculus cholecystitis in anergic patients. There are no pathognomonic signs, the diagnosis depends on suspicion of tuberculosis, peroperative findings and histological examination.**

*Keywords:* Gallbladder, tuberculosis

### INTRODUCTION

The gall bladder is an unlikely gastrointestinal organ to develop primary tuberculosis. There are no previous reports of series of gallbladder tuberculosis in cholecystectomy specimens though anacdotal reports in English literature are available since 1972 (Bargdahl and Boquist, 1972 [1]; Abascal *et al.*, 1988 [2]; Hahn *et al.*, 1995 [3]; Jain *et al.*, 1995 [4]; Cacciarelli *et al.*, 1998 [5]; Goyal *et al.*, 1998 [6]; Gupta *et al.*, 1998 [7]). In non-english medical archives, only English translation of their abstracts are available for review, one to two case reports are recorded during the same period. (Vojtistek and Zrustora, 1965 [8]; Burakov, 1965 [9]; Pfiefer and Reiher, 1966 [10]; Tasev, 1967 [11]; Krejezy and Krezezy, 1967 [12]; Mlynek and Engel, 1968 [13]; Erdelyi and Fenyvesi, 1969 [14]; Kretschmar and Rosenkranz, 1969 [15]; Mirki and Zeilnski, 1972 [16]; Ziarek *et al.*, 1973 [17]; Ziarek *et al.*, 1975 [18]; Ziarek *et al.*, 1975 [19]; Luez *et al.*, 1979 [20]; Czerwinski, 1979 [21]; Erokhmam *et al.*, 1982 [22]; Grassimi *et al.*, 1984 [23]; Piper *et al.*, 1987 [24]; Nakajo *et al.*, 1988 [25]; Strelis *et al.*, 1989 [26]; Arlandis *et al.*, 1990 [27]; Henriquez *et al.*, 1992 [28]; Ikai *et al.*, 1996 [29]; Jassem *et al.*, 1996 [30]; D'Agata *et al.*, 1997 [31]). Our 5 case reports of gallbladder tuberculosis, seen at Varanasi, Gorakhpur and Allahabad region of Eastern UP between the period 1995–98, is the largest till date.

### CASE REPORTS

1. Female, 26 year, presented with recurrent attacks of pain in the right hypochondrium. There was no history of jaundice, fever, weight loss or malena. Patient was anaemic

*Address for correspondence: 7 SPG Colony, Lanka, Varanasi - 221 005.

but not malnourished. US gallbladder showed cholelithiasis and cholecystitis. No mass lesion or lymph nodes were seen. Open cholecystectomy was done. The gallbladder was thick walled and it contained a single cholesterol stone. Histology of gallbladder showed tuberculosis of the wall. No other focus of tuberculosis was found.

2. Female, 45 year, presented with recurrent colicky pain in right hypochondrium. She had hysterectomy 4 years previously for a fibroid. US confirmed the clinical diagnosis of cholethiasis. Open cholecystectomy and histopathological examination of gallbladder revealed cholelithiasis and tuberculosis of the wall of gallbladder. There was no other overt focus of systemic or abdominal tuberculosis.
3. A 47 year old male patient presented with pain in the right hypochondrium. The pain was continuous with excecerbation related to meals. Clinical examination showed a firm globular enlargement of the gallbladder. The CT scan diagnosis was carcinoma of the gallbladder. Fine needle aspiration cytology examination was inconclusive. Through a sub-costal incision cholecystectomy was performed. Histopathological examination of the gallbladder showed it to be replaced with a tubercular mass. The postoperative recovery was complete.
4. Female, 21 year, presented with 3 years history of pain in right hypochondrium low grade fever and constipation. The pain was vague, dull and continuous. There was no vomiting. US examination revealed a 1.5 cm calculous and gallbladder wall thickness of 6 mm. Cholecystectomy through midline incision was carried out. Mesenteric lymph node biopsy and appendicectomy was done. Histopathological examination revealed tuberculous cholecystitis and mesenteric lymph node showed reactive hyperplasia. Liver and endometrial biopsy was negative for tuberculosis.
5. Female, 50 year, presented with pain right lower abdomen, irregular bowel habit and distension of abdomen for one month. Physical examination was noncontributory. US examination revealed small contracted gallbladder, single 1 cm stone and irregular thickness of wall. Laparotomy was carried out and cholecystectomy was done. The ileocaecal area was thickened. Histopathological examination of gallbladder revealed tubercular cholecystitis, cystic lymph node was replaced with caseaous material and biopsy of fibrofatty tissue at the ileocaecal area showed tubercular granuloma. In all the 5 patients anti-tubercular treatment (Rifampicin 450 mg, Isoniazid 3–5 mg/kg body weight, Parazinamide 22–35 mg/kg body weight) was given. The follow-up varied from 1–3 years. The symptoms were relieved in all cases.

The diagnosis of tubercular was on histopathological finding of caseaous necrosis.

### DISCUSSION

In the last 4 decades the incidence of gallbladder tuberculosis has shown a surge coinciding with re-emergence of abdominal tuberculosis, eight case reports are available for the period 1961–70, 7 each for 1971–80 and 1981–90 and for the period 1991–99, 10 case reports have appeared. The gallbladder is infected by mycobacterium tuberculosis as a part of miliary tuberculosis, abdominal tuberculosis or through the enterohepatic route. Four distinct clinical varieties of gallbladder tuberculosis are recognised [24]: (1) As a component of miliary tuberculosis in children and in adults, (2) As a component of disseminated abdominal tuberculosis [24, 16], (3) Isolated gallbladder tuberculosis without overt tubercular foci elsewhere in the body and (4) Involvement of gallbladder in anergic states due to uraemia [24], cancer [30] or aids [5]. Majority of the case reports are of isolated gallbladder tuberculosis but if post mortem is carried out multiorgan involvement may be found [28] in more cases. Tuberculosis of bile ducts producing stricture and dilatation [2] may progress to involve gall bladder, lymph node and adipose tissue in the portal tract [27]. Gall bladder harbouring benign lesions are prone to develop tuberculosis. Such association is reported for gallstone [31] diffuse papillomatosis of gallbladder [25] and opisthorchiasis [26]. In some patients the only evidence of gall bladder tuberculosis is on histological examination of the gall bladder [22] specimen after cholecystectomy. Most of 60 cases of gallbladder tuberculosis reported till 1987 were found on post mortem examination.

There is no pathognomonic presentation of gall bladder tuberculosis, it can vary from a surprise, on histological examination [22], to gall bladder perforation [3]. Gallbladder tuberculosis presents with symptoms of tuberculosis such as malaise, anorexia, low grade fever upper abdominal pain jaundice [2, 27], discharge at the umbilicus due to tuberculous seedling [6] gallbladder perforation and intrahepatic biloma [3]. In patients with anergic states due to AIDS acalculus choelcystitis occurs due to tuberculosis and the mycobacterium is found in the bile [5]. In one patient with aids intracellular mycobacterium avium was found in the wall of the gallbladder [5]. There are no specific investigations for gall bladder tuberculosis. Ultrasound examination [4] may show stone, wall thickness or associated liver lesion. Associated abdominal tuberculosis may indicate gallbladder involvement. Suspicion of gallbladder tuberculosis would lead to more cases being diagnosed and gallbladder tuberculosis should now form a routine differential diagnosis of gall bladder disease.

### *References*

[1] Bergdahl, L. and Boquist, L. (1972). Tuberculosis of gall bladder. *British Journal of Surgery*, **59**(4), 289–92.

[2] Abascal, J., Martin, F., Abreu, L., Pereira, F., Herrera, J., Ratia, T. and Menendez, J. (1988). A typical hepatic tuberculosis presenting as obstructive jaundice. *American Journal of Gastroenterology*, **83**(10), 1183–6.

[3] Hahn, S. T., Park, S. H., Shin, W. S., Kim, C. Y. and Shinn, K. S. (1995). Gallbladder tuberculosis with perforation and intrahepatic biloma. *Journal of Clinical Gastroenterology*, **20**(1), 84–6.

[4] Jain, R., Sawhney, S., Bhargava, D. and Berry, M. (1995). Gallbladder tuberculosis: Sonographic appearance. *Journal of Clinical Ultrasound*, **23**(5), 327–9.

[5] Cacciarelli, A. G., Naddaf, S. Y., el-Zeftawy, H. A., Aziz, M., Omar, Ws., Kumar, M., Atay, S., Abujudeh, H., Gillooley, J. and Abdel-Dayem, H. M. (1998). *Clinical Nuclear Medicine.*, **23**(4), 226–8.

[6] Goyal, S. C., Goyal, R., Malhotra, V. and Kaushik, K. (1998). Tuberculosis of the gallbladder. *Indian Journal of Gastroenterology*, **17**(3), 108.

[7] Gupta, N. M., Khaitan, A., Singh, V. and Radotra, B. (1998). Isolated gallbladder tuberculosis with post-operative biliary fistula. *Endoscopy*, **30**(6), 573–4.

[8] Vojtosek, V. and Zrustova, M. (1965). Tuberculosis of the gallbladder and its lymph nodes. *Lyon Chirorgical*, **61**(6), 828–37.

[9] Burakov, G. G. (1965). Tuberculosis of the gallbladder. *Problemy Tuberkuletza*, **43**(9), 79–80.

[10] Pfeifer, K. and Reiher, P. (1966). Tuberculosis of the gallbladder. *Deutsche Gesundheitswesen*, **21**(31), 1448–51.

[11] Tasev, Kh. (1967). Contribution to the cases of gallbladder. *Khirurgiia.*, **20**(2), 154–5.

[12] Krezczy, K. and Krejczy, H. (1967). A case of tuberculosis of the gallbladder. *Grizlica I Choroby Pluc.*, **35**(5), 491–2.

[13] Mlynek, H. J. and Engel, C. (1968). Contribution to the clinical aspects and morphology of gallbladder tuberculosis. *Zentrablatt fur Chirurgie.*, **93**(38), 1332–6.

[14] Erdelyi, B. and Fenyvesi, E. (1969). Isolated tuberculosis of the gallbladder. *Orvosi Hetilap.*, **110**(33), 1934–7.

[15] Kretschmar, K. H. and Rosenkranz, M. (1969). Gallbladder tuberculosis. *Zentrablatt Fur Allgemeine Pathologie Und Pathologische Anatomie.*, **112**(3), 281–5.

[16] Mirski, Z. and Zielinski, A. (1972). Isolated tuberculosis of the gallbladder and miliary tuberculosis of the liver discovered by means of biopsy. *Gruzlica I Choroby Pluc.*, **40**(6), 561–2.

[17] Ziarek, S., Deddoouche, M., Zoubir, Y. and Taleb, M. (1971). Tuberculosis of the gallbladder. *Tunisie Medicale.*, **51**(4), 217–9.

[18] Ziarek, S., Deddoouche, M., Zoubir, Y. and Taleb, M. (1975). Gallbladder tuberculosis in the light of our cases. *Polski Tygodnik Lekarski.*, **30**(26), 1113–5.

[19] Ziarek, S., Deddoouche, M., Zoubir, Y. and Taleb, M. (1975). Tuberculosis of the gallbladder. Apropos of 3 cases. *Medecine et Chirurgie Digestives*, **4**(4), 219–22.

[20] Luez, J., Deregnaucourt, G. and Benoit, M. (1979). Tuberculosis of the gallbladder. *Lille Medical*, **24**(2), 97–9.

[21] Czerwinski, L. (1979). Isolated gallbladder tuberculosis. *Polski Przeglad Chrirurgiczny.*, **51**(2), 173–4.

[22] Eroukhmanoff, P., Bazelly, B., Cohen-Solal, J. L., Milleron, B., Favre, M. and Flabeau, F. (1982). Tuberculosis of the gallbladder: Apropos of a case with histological identification. *Nouvelle Presse Medicale.*, **11**(51), 3795–6.

[23] Grassini, M., Cavanenghi, D., Amerio, G. M. and Sorisio, V. (1984). Apropos of a case of tuberculosis of the gallbladder. *Minerva Chirurgica.*, **39**(7), 573–5.

[24] Piper, C., Gamstatter, G., bettendorf, U. and von Egidy, H. (1987). Gallbladder tuberculosis. Review and case report of a patient with advanced renal failure. *Leber, Magen, Darm.*, **17**(6), 381–2, 385–6.

[25] Nakajo, S., Yamamoto, M., Urushihara, T., Kajitani, T. and Tahara, E. (1988). Diffused pappillomatosis of the gallbladder complicated with tuberculosis. *Acata Pathologica aponica.*, **38**(11), 1473–80.

[26] Strelis, A. K., Liadunkin, I. E. and Zadorozhnii, A. I. (1989). Diagnosis of disorders of the bile excretory system in patients with tuberculosis in association with chronic opisthorchiasis. *Problemy Tuberkuleza*, (1), 74–5.

[27] Arlandis Felix, F. J., Villalobos Talero, J., Mazure Lenhoff, R. A., Cabrerizo Comitre, E., Polo Camacho M., Bravo Arenzaa and Domiguez Garcia, M. (1990). Tuberculosis of the biliray system: presentation of a case and review of the literature. *Revista Espanola de Enfermedades Digestivas.*, **78**(1), 43–5.

[28] Henriquez, M., Trejo, C., Ojeda, M. and Benavides, A. (1992). Tuberculosis of the pancreas, an anatomoclinical case. *Revista medica de Chile*, **120**(10), 1153–7.

[29] Ikai, M., Itoh, M., Sano, H., Joh, T., Yokoyama, Y. and Takeuchi, T. (1996). Tuberculosis of the gallbladder. *Ryoikibetsu Shokogun Shirizu.*, (9), 219–21.

[30] Jassem, E., Smialek, U., Wojcikiewicz, K., Jaskiewicz, J. and Mierzejewska, E. (1996). Gallbladder tuberculosis and stomach cancer. *Pneumonologia I Alergologia Polska.*, **64**(12), 85–7.

[31] D'Agta, A., Funto, I., Lazzi, S. and Bonocompagni, G. (1997). Video laparoscopic cholecystectomy in a case of biliary lithiasis associated with gallbladder tuberculosis. *Minerva Chirurgica.*, **52**(9), 1103–8.

